# Teaching AI Ethics in Medical Education: A Scoping Review of Current Literature and Practices

**DOI:** 10.5334/pme.954

**Published:** 2023-10-16

**Authors:** Lukas Weidener, Michael Fischer

**Affiliations:** 1 UMIT TIROL – Private University for Health Sciences and Health Technology, Eduard-Wallnöfer-Zentrum 1, 6060 Hall in Tirol, Austria; 2 Head of the Research Unit for Quality and Ethics in Health Care, UMIT TIROL – Private University for Health Sciences and Health Technology, Austria

## Abstract

**Introduction:**

The increasing use of Artificial Intelligence (AI) in medicine has raised ethical concerns, such as patient autonomy, bias, and transparency. Recent studies suggest a need for teaching AI ethics as part of medical curricula. This scoping review aimed to represent and synthesize the literature on teaching AI ethics as part of medical education.

**Methods:**

The PRISMA-SCR guidelines and JBI methodology guided a literature search in four databases (PubMed, Embase, Scopus, and Web of Science) for the past 22 years (2000–2022). To account for the release of AI-based chat applications, such as ChatGPT, the literature search was updated to include publications until the end of June 2023.

**Results:**

1384 publications were originally identified and, after screening titles and abstracts, the full text of 87 publications was assessed. Following the assessment of the full text, 10 publications were included for further analysis. The updated literature search identified two additional relevant publications from 2023 were identified and included in the analysis. All 12 publications recommended teaching AI ethics in medical curricula due to the potential implications of AI in medicine. Anticipated ethical challenges such as bias were identified as the recommended basis for teaching content in addition to basic principles of medical ethics. Case-based teaching using real-world examples in interactive seminars and small groups was recommended as a teaching modality.

**Conclusion:**

This scoping review reveals a scarcity of literature on teaching AI ethics in medical education, with most of the available literature being recent and theoretical. These findings emphasize the importance of more empirical studies and foundational definitions of AI ethics to guide the development of teaching content and modalities. Recognizing AI’s significant impact of AI on medicine, additional research on the teaching of AI ethics in medical education is needed to best prepare medical students for future ethical challenges.

## Introduction

The use of artificial intelligence (AI) in medicine is expected to have a significant impact on patient care, medical research, and the entire healthcare system. The term ‘artificial intelligence’ was first coined in the 1950s by McCarthy et al. as ‘… the basis of the conjecture that every aspect of learning or any other feature of intelligence can in principle be so precisely described that a machine can be made to simulate it’ [1]. AI is typically associated with the field of computer science and can be divided into the so-called ‘strong AI’ and ‘weak AI’ [2]. Strong AI aims to develop a general AI with capabilities comparable to that of humans, while weak focuses on creating systems that can perform very specific tasks [3]. Weak AI is further divided into ‘symbolic AI’ and ‘statistical AI’, each with distinctive approaches to problem-solving and data analysis. While ‘symbolic AI’ is commonly based on predefined rules, currently used as expert-based clinical decision support systems (CDSS) in medicine for example, ‘statistical AI’ includes the field of ‘machine learning (ML)’, where numeric functions are used to establish patterns and correlations from the data [3].

Because of the ability to precisely analyze and process large amounts of data, applications based on ML are being successfully used in various medical fields, such as radiology, pathology, or dermatology [4,5,6,7]. Various other benefits are expected from the use of AI, such as increased accuracy in diagnosis, the possibility of personalized treatments, or a reduction in the workload of medical staff [7,8,9]. A more recent application of AI based on machine learning are large language models (LLM), which form the core of AI-based chat applications such as ChatGPT by OpenAI. First released to the public in November 2022, ChatGPT can be considered as the first consumer-grade and broadly used AI application [10]. Owing to the capabilities of ChatGPT in the medical field, for example, being able to pass the written portion of the US medical licensing examination (USMLE) [11], AI-based chat applications are expected to have a significant impact on medical practice [10,12].

One use case of symbolic AI in medicine is CDSS, which are applied to recommend treatment options based on predefined rules and expert knowledge [13]. Moreover, the use of AI in medicine is expected to reduce health-related expenditures and improve the accessibility of health services for patients [8,14,15].

Despite the many potential benefits expected from the use of AI, possible disadvantages should not be neglected. In addition to essential questions of user liability in the event of any errors in the use of AI-based applications in patient care or the security of patient data, ethical challenges are expected to be the most important [16,17]. In this context, for example, there is the possibility of bias due to a lack of representativeness of the applications used to train AI, potentially leading to the under- or overtreatment of patients [17,18]. Both symbolic and statistical AI can contribute to these ethical challenges [18]. For instance, CDSS as part of symbolic AI rely on predefined rules and decision trees, that may carry over existing biases from the developers and experts used in the development process [18,19]. Similarly, statistical AI algorithms may amplify biases in the data on which they are trained, further reinforcing inequalities [20]. Ethical challenges also include issues related to patient autonomy, data privacy, and transparency [5,14,21].

Owing to the potential challenges posed by using AI in medicine, current research on the topic recommends consistent and early teaching of future users [22,23,24]. In addition to imparting knowledge and fostering an understanding of AI in general, teaching about the ethics of AI is broadly recommended [24,25,26]. Recent studies further suggest that medical students anticipate significant ethical challenges posed by AI in medicine [27,28]. To ensure the best possible education for medical students, knowledge of the current literature regarding the teaching of AI ethics as part of medical curricula is necessary.

### AI ethics definition

The inconsistency in the scientific definition of AI is also reflected in the attempt to define AI ethics [29]. The interdisciplinary nature of the field, involving computer science and philosophy as well as different associated schools of thoughts such as humanism or transhumanism, further complicates the issue [30].

One prominent definition of AI ethics was provided by Leslie in 2019, stating that ‘AI ethics is a set of values, principles, and techniques that employ widely accepted standards of right and wrong to guide moral conduct in the development and use of AI technologies’ [31]. Whittlestone et al. also emphasize the importance of principles and standards regarding the use and development of AI and define AI ethics as ‘the emerging field of practical AI ethics, which focuses on developing frameworks and guidelines to ensure the ethical use of AI in society (analogous to the field of biomedical ethics, which provides practical frameworks for ethical practice in medicine.)’ [32]. Both definitions are consistent with current scientific and governmental efforts, including the European Commission’s High-Level Expert Group on Artificial Intelligence, to develop AI ethics guidelines for the ethical implementation and use of AI [33,34,35].

The analysis of current guidelines indicates that the effort is significantly influenced by the fundamental principles of medical ethics formulated by Beauchamp and Childress [36]. In addition to the principles of autonomy, non-maleficence, beneficence, and justice, recurring themes of AI ethics are for example transparency, explainability, accountability, and fairness [37,38]. Despite the focus to define suitable principles and values for the development and use of AI, the guidelines lack a clear definition of AI ethics [38].

Given the broad definitions formulated by Leslie and Whittlestone et al., we propose a more specific definition of ‘medical AI ethics’ that can be used for teaching and implementing AI ethics in medical education. We define ‘medical AI ethics’ as *an interdisciplinary subfield of AI ethics concerned with the application of ethical principles and standards to the research, development, implementation, and use of AI technologies within the practice of medicine*. Our definition emphasizes the importance of ethical principles and standards, following current efforts, such as from the World Health Organization (WHO), and reflects the importance of the established principles of medical ethics from Beauchamp and Childress [36,39]. By providing a narrower definition, we hope to reduce the complexity and scope of the topic of AI ethics in medical education and promote consistency in scientific publications in this field.

Based on the significant ethical challenges expected from the use of AI in medicine and the associated demand for teaching AI ethics within medical education, this study aims to synthesize and comprehensively overview the existing scientific literature on the teaching of AI ethics in medical education. Specifically, the current AI ethics teaching content and methods will be explored and discussed, with the goal of identifying areas for future research and providing the necessary groundwork to enhance the education of medical students.

## Methods

The present research was conducted based on the PRISMA-ScR guidelines as well as the methodological guidance for scoping reviews (JBI methodology) [40,41]. PubMed, Embase, Scopus, and Web of Science databases were searched. The initial search period was limited to the last 22 years (2000–2022). No scoping review protocol was published in advance of the research. The publication and all associated research have been approved by the ethical committee of the UMIT TIROL – Private University for Health Sciences and Health Technology.

### Inclusion criteria

Only publications that could be found in the databases mentioned above were included in the search. Furthermore, only publications written in English or German and that had an accessible abstract were considered for analysis.

### Search strategy

The following Medical Subject Headings (MeSH) terms and keywords were used for the database search: ethic* AND artificial intelligence OR ai AND medical school OR medical education OR medical curriculum OR medical students.

### Study selection

After database searching and removal of duplicates using literature management software (Mendeley; Elsevier), the results were evaluated for thematic relevance. In this context, the title and abstract of the respective publications were assessed for their suitability. Unsuitable publications, for example, due to lack of thematic relevance, were excluded from further evaluation. For further estimation of the suitability of the publications, an assessment of the full text took place. If there was thematic relevance concerning the objective of the present research, the publications were included in the study.

### Data extraction

During data extraction, data from all included publications were transferred to a table using Microsoft Excel (version 16.66). For each publication, the following data were recorded: First author’s name, year of publication, title, study type, the rationale for teaching AI ethics as part of medical curricula, recommendations on potential teaching content, recommendations on teaching modalities, and integration of teaching into medical curricula.

### Updated literature search

Due to the release of AI-based chat applications such as ChatGPT, Bard, or Bing Chat, a new literature search was performed in July 2023 using the original search strategy to account for any additional scientific literature published until the 30^th^ of June.

## Results

**Figure 1:**
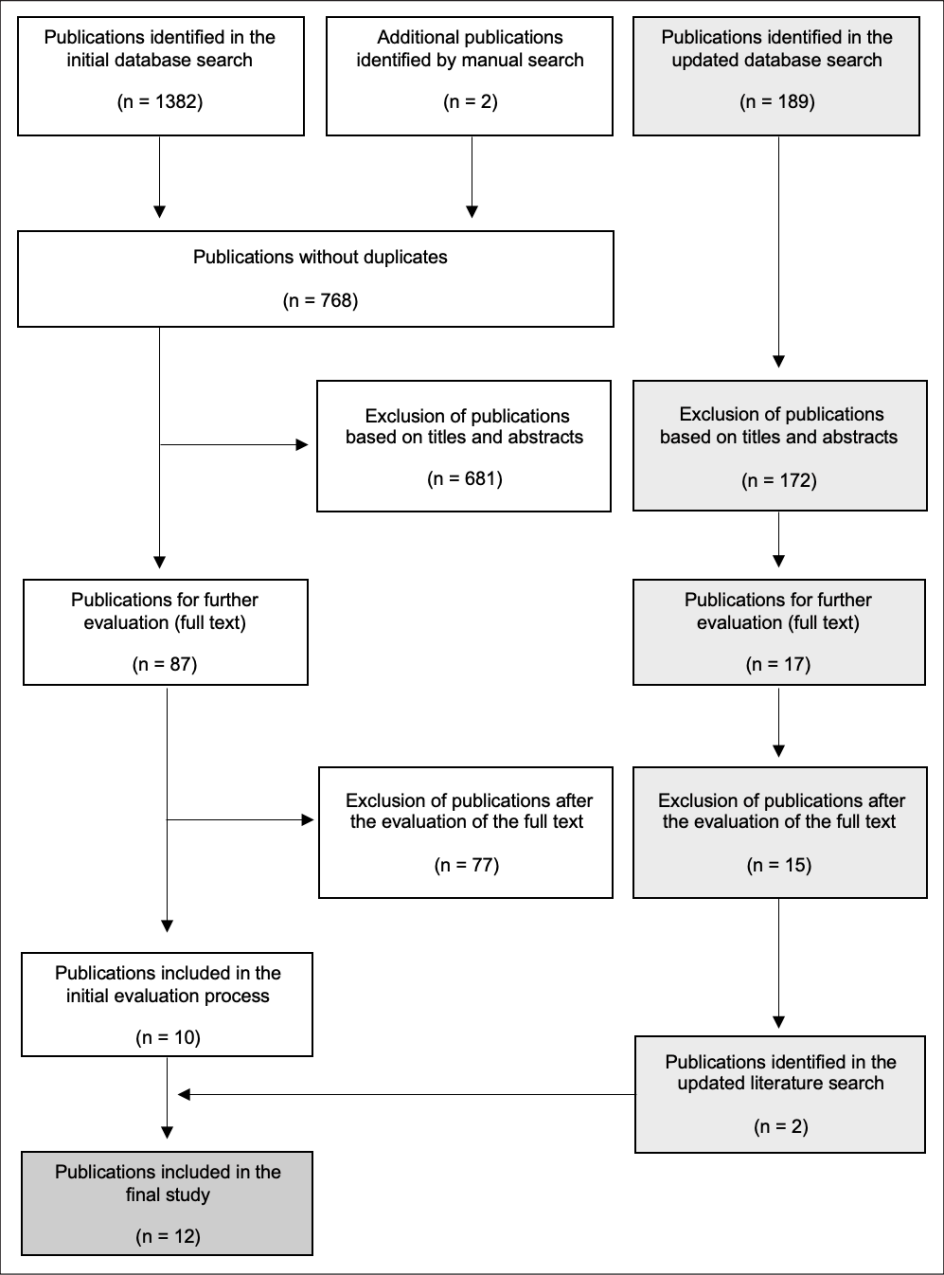
Flow chart of the search and selection process (adapted from PRISMA) [40].

The initial database search (January 2023) retrieved 1382 publications. By manual search, two additional publications were identified and included in the selection process. After removing duplicates (n = 616), and screening 10 publications remained. In the updated literature search (July 2023), 189 additional publications were identified. After reviewing the titles and abstracts, the full texts of 17 articles were further evaluated. Based on the prespecified exclusion criteria, 15 publications were excluded owing to a lack of thematic focus (e.g., publications regarding the impact of ChatGPT on medical education). Consequently, two additional publications were included in the subsequent analysis, resulting in a total of 12 publications. See Figure 1 for the flowchart of the search and selection process.

### Study characteristics

Despite the initial search period of 22 years (2000–2022), all publications included in the first analysis were published in the last four years (2018: n = 1; 2019: n = 2; 2020: n = 1; 2021: n = 4; 2022: n = 2). Two of the publications here were published by the same first author [42,43]. In addition to two reviews [43,44] and one cross-sectional study among medical students [45], commentaries or ‘viewpoints’ were the predominant study design among the included studies [22,23,42,46,47,48,49]. Except for two studies [47,49], the publications identified during the initial literature search focused on the integration and teaching of AI within medical curricula, without an emphasis on AI ethics. In the updated literature search two additional publications published in 2023 were identified and included into analysis. The included publications are presented in Table 1.

**Table 1:** Results of the literature search and selection process (included publications).


TITLE (YEAR)	METHODOLOGY	KEY FINDINGS

Grounded in reality: artificial intelligence in medical education (2023) [50]	Development, delivery, and assessment of an online, AI-integrated multidisciplinary course	The teaching of AI should receive dedicated time in the medical curriculum with a longitudinal approach, including preclinical and clinical education Ethics of AI (incl. Bias) are proposed to be taught in the clinical years

Commentary: The desire of medical students to integrate artificial intelligence into medical education: An opinion article (2023) [51]	Commentary	Systematic teaching of AI in medical education recommended to prepare future medical practitioners sufficiently Ethical principles regarding the use of AI in medicine and the associated data collection, storage and analysis should be taught within medical education.

Needs, Challenges, and Applications of Artificial Intelligence in Medical Education Curriculum (2022) [42]	Viewpoint	Due to the advancements of AI in medicine, AI should be implemented into the medical curriculum Interdisciplinary research is needed on how to implement AI into medical education To effectively address the broad ethical challenges introduced by AI in healthcare, instructors should possess a strong competency in bioethics

Artificial intelligence in medical education: a cross-sectional needs assessment (2022) [45]	Cross-sectional multi-center study	The current education on AI in medical education is limited The participating medical students perceived AI as an important topic for their medical education AI education should facilitate an understanding of AI ethics

The need for health Al ethics in medical school education (2021) [47]	Reflection	An understanding of the ethical challenges related to the use of AI in medicine is crucial to prepare medical students for upcoming challenges of medical practice The teaching of AI ethics should be based on ethical challenges such as informed consent, bias, safety, transparency, patient privacy, and allocation Real-life examples and case studies should be used to teach AI ethics

Readying Medical Students for Medical AI: The Need to Embed AI Ethics Education (2021) [49]	Viewpoint	AI ethics teaching should be based on associated ethical issues (e.g., bias) Teaching should align with existing medical ethics lessons Technical knowledge be taught as part of ethical lessons, while educating both academic staff and medical students

Educating Future Physicians in Artificial Intelligence (AI): An Integrative Review and Proposed Changes (2021) [43]	Integrative review	Although the teaching of AI within medical education is recommended, there are only few implementations reported Research is needed on how to best implement AI into medical curricula Medical students and future physicians should receive educations on the emerging ethical challenges related to the use of AI in medicine

Artificial Intelligence in Undergraduate Medical Education: A Scoping Review (2021) [44]	Scoping review	Medical education should prepare learners for the potential changes of the use of AI in medicine Lack of consensus on teaching modalities and content related to AI identified Curricular content on AI and AI ethics recommended

What do medical students actually need to know about artificial intelligence? (2020) [22]	Commentary	Curricular and extracurricular learning opportunities on AI technologies should include ethical implications of AI AI education should be available after medical school

Reimagining Medical Education in the Age of AI (2019) [23]	Viewpoint	Medical education should help medical students to respond to the ethical challenges that arise due to the use of AI in medicine Empathy and compassion should be fundamental to the curricular development and teaching related to AI

Introducing Artificial Intelligence Training in Medical Education (2019) [46]	Viewpoint	The topic of AI and related content should be part of the medical curriculum, with a staged approach throughout medical education Preclinical education should include ethics and legal issues with AI

Machine learning and medical education (2018) [48]	Perspective	Machine learning (ML) and data science should be part of medical education Risks, benefits and ethical issues related to the use of ML in medicine should be taught.


### The rationale for teaching ethical aspects of AI as part of medical education

There is unanimity among the authors regarding the expected significant impact that the use of AI in medicine will bring. The expected significant impact of AI in medicine also brings various ethical challenges, such as the potential loss of empathy in the doctor-patient relationship and changes in the structure of trust [42,45]. Given the lack of guidelines on the use of AI in medicine and the associated ethical challenges, it is recommended that medical education consistently include teaching on the ethical aspects of AI [43,46,51]. The potential for bias due to unrepresentative data and the resulting disadvantage for certain populations is a frequently cited reason for the need to integrate or expand the teaching of AI ethics in medical education, as well as recommended teaching content [22,42,45,47,49,50].

### Recommendations for teaching content

Four publications do not specify possible teaching content for AI ethics [43,46,48,50]. Instead, three of the four publications propose broadly addressing general ethical problems and challenges that the use of AI in medicine may pose [43,46,48]. The authors of these publications do not provide further information about their definition of general ethical problems or challenges [43,46,48]. While a discussion on ethics regarding the use of AI is proposed to be taught in the clinical years in one of these publications there was no further specification [50].

In contrast, half of the included publications recommend teaching data protection and its potential impact on patient care when using AI in clinical practice [22,44,45,47,49,51]. Furthermore, four publications emphasized the importance of teaching the ethical aspects of user liability when AI is used in a clinical context [22,44,45,49].

Wartman et al. highlight empathy as a cornerstone of teaching and curriculum development on AI [23]. Rethinking the teaching of ethics is recommended to prepare medical students for the complex ethical issues that may arise between patients, caregivers, and AI [23]. The anticipated ethical implications of using AI in medicine, including issues related to bias and patient and physician autonomy, are identified as the basis for developing detailed teaching content on AI ethics in three of the included publications which will be further examined in more detail [22,47,49].

In their publication, McCoy et al. recommend the promotion of an understanding of fairness, transparency, and responsibility regarding the use of AI, similar to the established principles of medical ethics outlined by Beauchamp and Childress, including beneficence, justice, autonomy, and non-maleficence [22,36]. Four of the 12 included publications also recommend these principles as the foundation for teaching AI ethics [44,45,47,49].

Two publications that focus on teaching ethics on AI as part of medical education were identified. Katznelson et al. not only illustrate the relevance of teaching ethics on AI but also present six specific ethical challenges (‘informed consent’, ‘bias’, ‘safety’, ‘transparency’, ‘patient privacy’, and ‘allocation’) that should be addressed as part of medical school teaching and student training [47]. Quinn et al. also echo the possible teaching content defined by Katznelson et al. on the ethics of AI as part of medical school teaching and illustrate its relevance. In addition, Quinn et al. cite other possible teaching content based on fundamental ethical challenges and issues that may arise from the use of AI such as the ethical issues that may arise from overreliance on AI by users and potential interference with patient autonomy [49]. Quinn et al. further emphasize the need to understand the impact of AI on existing basic principles of medical ethics. Teaching as part of medical curricula should continue to address the ethical aspects that may arise from incorrect, absent, or abusive use of AI in medicine [49].

Table 2 provides a more detailed overview of the recommended teaching content for AI ethics in medical education identified in the publications reviewed.

**Table 2:** Recommended artificial intelligence (AI) ethics teaching content.


MAIN TEACHING RECOMMENDATION	DETAILED AI ETHICS TEACHING CONTENT RECOMMENDATIONS	PUBLICATIONS

Ethical challenges and issues^a^	General ethical problems and challenges (not specified) [43,46,48,50] Informed consent, bias, safety, transparency, patient privacy, and allocation [47,49] Quality assurance, trust, patient values, confidentially, justice, human rights, accountability, over-diagnosis and over-treatment, automation bias, skill erosion, explainability, and information overload [49] Incorrect, absent, or abusive use of AI in medicine [49] Patient-physician relationship [22,42,45]	[22,42,43,45,46,47,48,49,50]

Data protection^a^	Cyber-security risks [49] Risks for patient data due to the use of AI [22,44,45,47] AI-related data collection, storage, and analysis [51]	[22,44,45,47,49,51]

Liability^a^	Liability in case of mistakes due to programming or construction flaws, lack of proper documentation, and user guidance [49] Ethical implications of liability in the clinical context (not specified) [42,44,45,46]	[22,44,45,46,49]

Ethical values and principles^a^	The potential impact of AI on the principles of medical ethics by Beauchamp and Childress (beneficence, justice, autonomy, and non-maleficence) [22,36,42,44,45,49] Fairness, transparency, and responsibility analogous to beneficence, justice, autonomy, and non-maleficence [22] Empathy as the cornerstone of teaching AI ethics [23]	[22,23,42,44,45,47,49]


^a^associated with the use of AI in medicine.

### Recommendations on teaching modalities and integration of teaching content

Recommendations on teaching modalities and the procedure for integrating the teaching of ethical aspects of AI vary based on the included publications [43,48]. Wartman et al. advocate for a major overhaul of current medical curricula, emphasizing empathy and compassion and focusing on knowledge management rather than information acquisition and retention [23]. This approach would involve a fundamental and radical rethinking of teaching in medicine to prepare for the expected impact of AI on the field [23]. Quinn et. Al present a much less disruptive approach, which is intended not only to allow the integration of teaching ethical aspects of AI without significant changes to existing medical ones, but also to allow simultaneous teaching of ethical and technical backgrounds to AI [49]. These authors propose four steps to integrate AI ethics teaching into medical curricula [49]. In the first step, ‘formulation’, teaching content should be defined based on potential ethical problems and challenges. In the second step, ‘readying lessons’, previously defined teaching content needs to be aligned with existing modules in the field of ethics. ‘readying staff’, the third step, provides ethics instructors with the technical knowledge to effectively communicate potential ethical issues and challenges posed by AI. The fourth and final step, ‘readying students’, is intended to teach medical students. The presented approach is expected to allow a timely integration of teaching on the ethics of AI without often necessary and costly accreditation processes that would require a complete restructuring of medical curricula [49]. While Quinn et al. present concrete steps for integrating the teaching of AI ethics in their publication, there is no specification of possible teaching modalities concerning the respective teaching units.

Case-based teaching using real-world examples of AI in clinical contexts and the associated ethical challenges is recommended in five publications [22,42,45,46,47]. In this context, three of the publications recommend teaching in small groups as well as interactive-oriented seminars [22,44,45]. Linking theoretical teaching with practical self-application of AI is a preferred teaching method in three of the publications [22,45,48]. While one of the two publications that were identified throughout the updated literature research lacks specification on teaching modalities or the integration of AI ethics teaching content [51], the ethics of AI is proposed to be taught in the clinical years based on discussions in the second one [50].

## Discussion

This scoping review synthesizes the current literature, largely comprising commentaries and viewpoints, on AI ethics in medical education, with a focus on 12 publications published within the past five years (2018 – 2023). Compared to recent reviews on the teaching of AI as part of medical curricula, only a reduced number of publications could be included, due to a narrower focus on AI ethics [24,43,44]. Of the 12 publications included, only two specifically focused on the teaching of AI ethics in medical curricula [47,49]. The remaining ten publications emphasized the relevance of AI ethics in medical education but varied in their specification of possible teaching content and modalities [22,23,42,43,44,45,46,48]. Both publications which focused on teaching AI ethics within medical education were published in 2021, which, in addition to recent research interest, may also imply limited awareness within the scientific community [47,49].

### Recommended teaching content

This review highlights the high need for research regarding the teaching of ethics in the aspect of AI as part of medical education. Although all publications included in the evaluation emphasize the relevance of teaching the ethics of AI, possible teaching was only concretized in three publications [43,46,48]. While the lack of concretization might not only be attributable to the divergent focus on the general integration of AI within the scientific community, but rather due to the missing content on AI ethics in general [35,52].

The inconsistency in the definition of AI within the evaluated publications further limits the comparability of current literature and scientific research efforts regarding the definition of AI ethics-related teaching content [43,44,45]. As the authors’ understanding of AI and AI ethics is crucial to interpret the results of the respective studies, such as the recommended teaching contents, this review highlights the need for a common and clear understanding of these terms in the context of medical education. This requires disclosing the knowledge and understanding that the publications are based on. Out of 12 publications included in this review, only one provided a definition of AI ethics [47].

The publication by Katznelson et al. (2021) was identified as the first to not only focus on the definition of AI ethics and the integration of AI in medical education but also to formulate specific recommendations for teaching content by anticipating ethical challenges and problems that may arise from the use of AI in medicine [47]. Similarly, Quinn et al. (2021) defined anticipated ethical challenges as the foundation of potential teaching content [49].

The principles of medical ethics defined by Beauchamp and Childress are widely cited as the essential foundation of any teaching content [36,44,45,47,49]. While utilizing expected ethical challenges from AI in medicine, and Beauchamp and Childress’s principles of medical ethics, as a foundation for AI ethics teaching content seems beneficial, the practicability and implications for teaching AI ethics have yet to be assessed in subsequent research.

The inconsistency of the publications regarding possible teaching content on AI ethics, as well as the general definition of AI and AI ethics highlight the significant need for further research in the field. However, the definition of medical AI ethics presented in this review can serve as a basis for establishing more uniform teaching content on AI ethics. By addressing the recommendations from the authors of the publications included in this review, the definition reflects the growing importance of ethical considerations in the development and use of AI in medicine.

### Recommended teaching modalities

The authors’ recommendations for the best possible integration of teaching on the ethics of AI range from a fundamental restructuring of medical school teaching [22] to an integrative teaching of technical and ethical content on AI without the need for significant changes to existing curricula [49].

The publication by Quinn et al. was the only publication that could be identified, presenting a concrete concept regarding the teaching and integration of AI ethics within medical education [49]. A widely anticipated difficulty in teaching AI ethics, or AI in general, as part of medical education, is the lack of sufficient teaching staff or knowledge on the part of existing teaching staff [42,46,49,50,51,53,54]. Other significant challenges include overloaded medical curricula with no dedicated time for AI ethics, and a lack of established standards for this emerging field of study [49,50]. Although the complexity of AI and the novelty of the field of AI ethics seem to be leading factors regarding the difficulty to find sufficiently trained teaching staff, without standardization in terms of AI ethics and related teaching content, the preparation of teaching staff and students will be significantly more difficult. While establishing clear terminology regarding AI, AI ethics, and teaching content can contribute to a shared academic understanding, it may not directly address the current limitations of faculty expertise in this area. This lack of expertise can likely be linked to the novelty of AI in medical education and the rapidly evolving nature of the field. Furthermore, the current lack of consistency in teaching AI ethics, often viewed as a challenge, may also reflect the necessary diversity native to ethics. This diversity allows for a range of perspectives and facilitates interdisciplinarity, which could be particularly valuable in such a rapidly evolving field. Nonetheless, reaching some level of agreement on the core elements of AI ethics and related teaching content, at least at the national level, could contribute to the comparability and standardization of medical education beyond individual institutions.

Five of the evaluated publications recommend case-based learning, which aligns with the current efforts for competence-oriented and evidence-based teaching in medical education [22,42,45,46,47,55,56]. Furthermore, the evaluated publications recommend the integration of practical and theoretical teaching content on AI ethics as well as teaching in interactive small groups and seminars [22,45,48]. Given the emphasis on case-based teaching by nearly half of the publications, the lack of concretization on possible examples related to teaching AI ethics becomes imminent [42,43,46].

Analogous to the heterogeneity of the results regarding the recommended teaching modalities of AI ethics, the question of who is ideally qualified to instruct AI ethics remains largely undetermined.

Quinn et al. proposed the idea of incorporating the technical aspects of AI into the medical ethics segment of the curriculum, where ethicists, once trained in the basics of AI, would serve as educators [49]. Additional research is required to assess the viability of this approach in an educational setting and to determine whether traditionally trained ethicists could adequately deliver not only content on AI ethics but also general instruction on AI in medicine.

Further divergence exists regarding the optimal time for implementing AI ethics into the medical education curriculum. While the majority of authors do not explicitly suggest when AI instruction should be implemented, there are varied opinions among those who do. Some propose introducing AI ethics in the preclinical phase in line with the conventional schedule for medical ethics instruction [43,44,46,49], while others advocate for its introduction during the clinical years, reasoning that students would have a better comprehension of the potential challenges and issues at this later stage [50].

### Updated literature search

In response to the release of AI-based chat applications, such as ChatGPT, in November 2022, an updated literature search was conducted identifying two new publications [50,51]. Although both of these works were published after November 2022 and, thus, after the launch of ChatGPT, neither specifically mentions AI-based chat applications or ChatGPT. Both publications underscore the significance of ethics in the context of AI usage in medicine, with references to potential bias [50] and ethical principles concerning AI data utilization [51]; however, they fail to provide a clear definition of AI ethics or specific teaching content. The lack of precise recommended teaching content in these two newly identified publications aligns with the results of the initial literature search. For instance, while Krive et al. proposed a four-week elective course on AI in medicine to be incorporated into the preclinical years of medical education, they suggested teaching AI ethics in clinical years without providing specifics.

During the updated literature search, articles discussing the use of AI-based chat applications, such as ChatGPT, in medical education were discovered. Although these publications did not meet the predefined selection criteria due to insufficient focus on AI ethics, they underscored the anticipated substantial impact of AI-based chat applications on medical education [57,58,59]. The adoption of AI-based chat applications, such as ChatGPT, in medical education highlights the need for additional research on AI ethics in this context [59]. Notably, unlike medical products or applications specifically developed for use in healthcare, which must comply with ethical standards and principles during their development and implementation processes, AI-based chat applications such as ChatGPT do not need to comply with the same strict and formal requirements, as they are not explicitly designed for medical use. Therefore, the utilization of ChatGPT and similar AI-based chat applications by medical students during their education poses novel ethical challenges [59]. These include, but are not limited to, the transparency and explainability of the provided medical information and the accessibility of the applications [58]. This further emphasizes the necessity for future physicians to be acutely aware of the ethical challenges inherent to AI utilization in medicine. This awareness should be fostered during their education to prepare them to navigate this increasingly complex landscape.

### Limitations

The study’s limitations include a limited number of search terms and databases used, which may have resulted in missing relevant publications. Additionally, only publications written in English were included in the evaluation (no publications in German could be identified). Furthermore, only 12 publications were identified in the literature search, with only two publications focusing on teaching AI ethics in medical education [47,49]. While an updated literature search was performed to account for the release of AI-based chat applications, such as ChatGPT, the results of this study are likely to be subject to extensive changes due to the rapid developments in AI technologies.

## Conclusion

This scoping review aimed to synthesize and present the current scientific literature on the teaching of AI ethics in medical education. The findings underscore the recency, theoretical nature, and scarcity of the available literature on this topic. AI is predicted to significantly impact medicine, which makes the teaching of AI ethics an indispensable part of medical education. However, there is a notable lack of empirical studies and evaluations of existing educational programs. Only two publications specifically focus on the teaching of AI ethics in medical curricula. These findings highlight an urgent need for further research, particularly empirical and practice-based studies, to successfully integrate teaching on the ethics of AI into medical curricula. Moreover, the results suggest that there is currently a lack of a foundational definition of AI ethics, which could be beneficial for guiding the creation of teaching content and modalities. Recognizing that such definitions will need to be adapted in response to advancements in AI technologies and our evolving understanding of the associated ethical implications, continuous dialogue and further research within the field will be essential.
